# Pluripotent Stem Cells: Recent Advances and Emerging Trends

**DOI:** 10.3390/biomedicines13040765

**Published:** 2025-03-21

**Authors:** Aline Yen Ling Wang, Ana Elena Aviña, Yen-Yu Liu, Huang-Kai Kao

**Affiliations:** 1 Center for Vascularized Composite Allotransplantation, Chang Gung Memorial Hospital, Taoyuan 333, Taiwan; 250783@cgmh.org.tw (A.E.A.); louis881128@cgmh.org.tw (Y.-Y.L.); 2 International PhD Program in Medicine, College of Medicine, Taipei Medical University, Taipei 110, Taiwan; 3 Department of Plastic and Reconstructive Surgery, Chang Gung Memorial Hospital, Taoyuan 333, Taiwan; kai3488@cgmh.org.tw; 4 College of Medicine, Chang Gung University, Taoyuan 333, Taiwan

The field of induced pluripotent stem cells (iPSCs) continues to evolve, offering unprecedented potential for regenerative medicine, disease modeling, and therapeutic applications. All 10 featured research papers collected in this Special Issue demonstrate the assessment of distinct iPSC features, which include molecular regulations and differentiation protocols, in addition to therapeutic applications, along with genetic editing practices and immune evasion developments. This editorial presents a summary of the essential discoveries within all published works from this issue and establishes their widespread field impact together with upcoming trends. (1) Advances in iPSC-based regenerative medicine by Jin et al. (2023) provides a review of iPSC-based treatments for urethral regeneration, analyzing the drawbacks of conventional graft procedures [1]. The paper by Du et al. (2023) examines stem cell-signaling in treating intervertebral disk degeneration, and demonstrates iPSC-derived therapies as a promising musculoskeletal disorder solution [2]. (2) Molecular and genetic insights in iPSCs by Machado et al. (2023) examines the molecular functions of OCT4 and SOX2 during iPSC reprogramming by exploring their effects on epigenetic modifications while discussing their role in keeping stem cells pluripotent [3]. Zhang et al. (2023) present an extensive review about CRISPR-Cas9 gene editing of iPSCs, which demonstrates both advanced genome precision techniques and promising medical possibilities [4]. Saini et al. (2023) examine ATP-binding cassette proteins together with their regulatory functions for sustaining stem cell pluripotency while demonstrating the importance of intracellular transport systems [5]. (3) Neural differentiation and neurological applications: Neural differentiation research combined with applications in neuroscience by Yarkova et al. (2024) develops a distinct approach to detect endoplasmic reticulum stress in induced pluripotent stem cell-derived neurons for helping model neurodegenerative diseases [6]. Research by Lee et al. (2024) demonstrates how iPSCs can differentiate into neurons, which shows encouraging results in preclinical studies for treating Parkinson’s disease [7]. (4) Machine learning and computational applications by Vedeneeva et al. (2023) presents machine learning technology that automatically detects iPSC colonies for high-quality selection enhancement while improving differentiation outcomes [8]. According to Chung et al. in (5) Developmental and signaling pathways (2024), BMP signaling controls the differentiation process of neural crest cells and ectodermal placode cells, which helps advance knowledge of embryological pathways using iPSC technology [9]. (6) Immune evasion strategies in iPSC-based therapies by Mu-u-min et al. (2025) presents immune evasion techniques for stem cell-based diabetes therapy as they review methods including encapsulation, along with genetic modifications to escape immune rejection difficulties [10].

The field of iPSCs has recently made significant improvements in safety together with efficiency and clinical practice readiness [11,12]. Modern reprogramming methods have reduced genomic alterations through the development of safer non-integrative approaches, including messenger RNA (mRNA) transfection and Sendai virus delivery, along with small molecule-based reprogramming to replace traditional viral methods for generating clinical-grade iPSCs [13,14,15,16]. The cells receive reprogramming factor genes OCT4, SOX2, KLF4, and c-MYC through transient mRNA transfection procedures, which transiently expresses these factors [13,15,17]. The genome-changing risks during reprogramming are minimized by mRNA transfection because it does not lead to integration. Through this method, scientists obtain controlled gene expression and faster reprogramming kinetics. Sendai virus delivery technique is another powerful tool for reprogramming [18,19,20,21]. It is replication-deficient and does not integrate into the host genome, making it safer for generating clinically relevant iPSCs. This method has been widely adopted for generating GMP-compliant (Good Manufacturing Practice) iPSCs for clinical applications. Researchers investigated reprogramming using chemical substances instead of classical transcription factors because these substitutes reduce mutation risks and enhance the process efficiency [22,23]. Simultaneously, the creation of 3D organoid models has taken the power of iPSCs past traditional differentiation methods to produce real-life models of brain and liver together with gastrointestinal system structures that help scientists examine diseases, test new drugs, and develop regenerative medical treatments [24,25,26]. Scientists can now use CRISPR-Cas9 in combination with iPSC technology to develop personalized regenerative treatments, thanks to recent field revolutionization [27,28,29]. For example, researcher access to disease mechanisms is enhanced through the gene-editing of iPSCs that come from patients with genetic disorders to produce matching control lines for scientific studies [29,30]. Parkinson’s disease-specific neurons derived from iPSCs allow researchers to edit them for disease-progression investigation of key genes [31]. Additionally, CRISPR-based technologies serve as tools to fix genetic errors found in iPSCs extracted from patients before converting these cells into healthy transplantation-ready cells [32,33]. Research has shown that autologous cell therapy could become possible through dystrophin gene correction in Duchenne muscular dystrophy patient-derived iPSCs [34]. Moreover, the latest CRISPR systems, such as base editors and prime editors, enable exact gene modification that produces fewer errors while avoiding double-strand break formation to minimize unintended mutations. Finally, gene-edited iPSCs are now being used to create humanized disease models for drug screening, allowing for the identification of compounds that target disease-specific pathways [35,36,37,38]. Additionally, refined differentiation protocols leveraging key signaling pathways, including BMP, Wnt, and TGF-β, have enhanced the efficiency and reproducibility of iPSC-derived cardiomyocytes, neurons, and pancreatic β-cells, accelerating their potential clinical applications [39,40,41]. However, the main challenge that prevents iPSC-based therapies from implementation is immune rejection. Research teams may employ CRISPR-Cas9 strategies to engineer hypoalloreactive iPSCs by removing HLA class I and II molecules and reducing immune surveillance, while adding PD-L1 regulatory proteins for developing universal cell supplies that would not require immunologic suppressing drugs [42]. Multiple technological developments, together, are accelerating the usage of iPSC technology for translation applications, which is leading medicine toward broad personalization and regeneration at a scale not seen before.

Several issues continue to challenge the advancement of iPSCs, even though research conducted in this Special Issue shows great potential for their use. For example, the obstacles to moving iPSC-derived cell products toward clinical implementation include maintaining reproducibility and scalability. The risk of genetic instability and tumor formation in iPSC-derived cell therapies necessitates stringent safety assessments. Because iPSCs are widely utilized in disease modeling and tissue transplantation methods, the existing ethical rules, together with regulatory procedures, must adapt properly, such as through the implementation of AI with machine learning capabilities in the field of stem cell research. Computational methods described by Vedeneeva et al. (2023) hold great potential to transform current processes for quality checks and cell differentiation protocols [8]. Applied translation of iPSC-based treatments for medical use continues as a key objective that depends on united work between experts in stem cell biology and bioengineering and medical sciences.

The studies assembled in this Special Issue deliver valuable research about pluripotent stem cells together with their potential medical applications. The data proves the versatile nature of iPSCs, as they continue to drive advances in both regenerative medicine and gene therapy together with disease modeling applications. The combination of gene editing along with bioengineering methods and computational technologies will enhance the development process for safe and effective iPSC-based therapeutic options.

As a Guest Editor of this Special Issue, we express heartfelt thanks to all the authors, together with reviewers and researchers who advanced the discipline. iPSC cells have only just begun their path, yet this path leads directly to a new age of regenerative healthcare that will rely heavily on these cells to define its future developments.
